# Resistance to acetylsalicylic acid in patients with type 2 diabetes mellitus is associated with lipid disorders and history of current smoking

**DOI:** 10.1007/s40618-013-0012-2

**Published:** 2014-01-09

**Authors:** B. Łabuz-Roszak, K. Pierzchała, K. Tyrpień

**Affiliations:** 1 Department of Neurology in Zabrze, Medical University of Silesia, 3-go Maja 13/15, 41-800 Zabrze, Poland; 2 Department of Chemistry, Medical University of Silesia, Zabrze, Poland

**Keywords:** Diabetes mellitus, Acetylsalicylic acid, Stroke, Aspirin resistance

## Abstract

**Background:**

Diabetes mellitus (DM) is an important risk factor for stroke. Acetylsalicylic acid (ASA) is the most frequently used medication for prevention of cardio-cerebral vascular diseases. However, some patients experience ischaemic vascular events despite the use of ASA. This phenomenon is known as “aspirin resistance” (AR). The aim of this study was to assess the prevalence of AR in diabetic patients and search for factors associated with it.

**Materials and methods:**

The examined group consisted of 96 subjects with diagnosed type 2 DM. Platelet function test was performed by the method of whole blood impedance aggregometry.

**Results:**

Among examined subjects, 51 patients (53.1 %) were sensitive to ASA action (ASA responders) and 45 patients (46.9 %) were resistant to ASA action (ASA non-responders). No association was found between platelet aggregation and gender, age, dose of ASA, known duration of diabetes, BMI, heart rate, mean systolic and diastolic blood pressure, and risk factors except for current smoking (*p* = 0.030). ASA non-responders were treated shorter with ASA than ASA responders (*p* = 0.010). The mean total cholesterol (*p* = 0.020), LDL concentration (*p* = 0.005), HCT (*p* = 0.010), WBC (*p* = 0.030), and PLT (*p* = 0.050) were significantly higher in ASA non-responders. No association was found between AR and results of other laboratory tests and medications. Multiple logistic regression analysis revealed factors associated with AR: current smoking and LDL concentration higher than 3.5 mmol/l.

**Conclusions:**

Results of our study did not confirm the association between poor glycaemic control in the diabetic patients and AR. Resistance to ASA in diabetic patients is associated with lipid disorders and history of current smoking.

## Introduction

Diabetes mellitus (DM) is an important and common risk factor for stroke. People with DM suffer from cerebrovascular events 1.5–3 times more likely than people without disorders of carbohydrate metabolism. Acetylsalicylic acid (ASA) is the most frequently used medication for prevention of ischaemic events in diabetic patients. Recommendations for the cardiovascular prevention and usage of ASA in diabetic patients are nowadays similar to the general principles as for people without diabetes. Recent randomized clinical trials and meta-analyses did not confirm the effectiveness of ASA in primary prevention in diabetic patients without other risk factors [1–4]. Following the current European guidelines [5], ASA is recommended in all patients with an overt vascular disease (previous stroke, previous non-cardioembolic cerebral transient ischaemic attack, coronary heart disease, previous myocardial infarction, peripheral artery disease). In asymptomatic people, also in diabetic patients, ASA could be considered when the risk of death from a cardiovascular disease is high (5 % or greater in the next 10 years). On the basis of the risk factors (gender, age, systolic blood pressure, total cholesterol and history of smoking), one can determine the risk of death from cardiovascular causes in patients aged 40 and older using tables of Systematic Coronary Risk Evaluation (SCORE) [5, 6].

In patients who regularly take ASA a 25 % reduction of the incidence of myocardial infarction and ischaemic stroke was reported [7]. However, some patients do not experience the positive antiplatelet effect and have cardiovascular events despite the use of ASA. This phenomenon is known as “clinical aspirin resistance” (AR). This term is also used when laboratory test results indicate the lack of antiaggregative ASA action (known as “biochemical aspirin resistance”).The prevalence of AR described in the literature is up to 60 % among subjects taking ASA for primary or secondary prevention of cardiovascular episodes [8–15]. Some authors noticed that AR occurred more frequently in diabetic patients [16, 17]. The mechanism of AR has not been fully understood as yet. Genetic factors are considered together with patient non-compliance, low dose of the drug, interactions with other medications and substances [18]. In diabetic patients the role of glycaemic control was also studied but the data concerning association between poor control and AR are conflicting [16, 17, 19–21].

The aim of this study was to assess the prevalence of AR in diabetic patients and search for factors associated with this phenomenon.

## Materials and methods

Subjects for the study were recruited in 2011–2012 years from patients treated in the Voivodship Outpatient Clinic for Diabetic Patients in Zabrze, Poland. The recruitment for the study was performed once a month. All the patients attending the outpatient clinic on the recruitment day were checked according to the inclusion and exclusion criteria. Only those who agreed to take part in the study and gave written consent were selected.

The inclusion criteria for the examined group were the following: diagnosed diabetes mellitus type 2, known cardiovascular disease (coronary heart disease, previous myocardial infarct, previous stroke or transient ischaemic attack, peripheral artery disease) or SCORE > 5 %, regular daily ASA intake at the dose of 75–150 mg/day, not using other antiplatelet and/or anticoagulant agents, age between 18 and 80 years, and the patient’s informed written consent for the study.

Diabetes mellitus was diagnosed according to the current criteria of Polish Diabetological Society (clinical symptoms of hyperglycaemia and casual glycemia ≥200 mg/dl or twice measured fasting glycemia ≥126 mg/dl or glycemia in 120 min of OGTT ≥ 200 mg/dl).

The 10-year risk of fatal cardiovascular disease (SCORE) was assessed using the following data: sex (male/female), age (years), systolic blood pressure (mmHg), total cholesterol (mmol/l) and history of current smoking [5, 6].

The exclusion criteria for the examined group were the following: the intake of other oral antiplatelet or anticoagulant agents, the use of other non-steroidal anti-inflammatory drugs (NSAIDs), irregular ASA intake, the use of heparin or low molecular weight heparin, platelet count <100 × 10^3^/μl or >450 × 10^3^/μl, history of haemorrhage.

Patients with history of atrial fibrillation participating in this study had contradictions for usage of oral anticoagulant agents or did not agree for such treatment.

The regularity of ASA intake (one tablet per day) was determined based on the carefully obtained medical history. Besides, the intake of ASA was personally controlled by one of the authors on the day (24 h) before the study.

The study was approved by the Bioethics Committee of the Medical University of Silesia.

All patients were interviewed and underwent physical examination. Arterial hypertension was recognized in people treated with antihypertensive drugs and in those who presented abnormal BP in two measurements (SBP ≥ 140 mmHg and/or DBP ≥ 90 mmHg). Dyslipidaemia was recognized in people treated with statins or fibrates and in those who revealed abnormal results of lipids (total cholesterol ≥ 4.5 mmol/l and/or LDL ≥ 2.6 mmol/l and/or TG ≥ 1.7 mmol/l). Coronary heart disease, previous myocardial infarct and atrial fibrillation were recognized only when they were documented.

A 10-ml fasting blood sample was obtained to determine platelet function and additional laboratory tests (CBC, glucose, total cholesterol, LDL, HDL, triglycerides, HbA_1_C, CRP, creatinine). CBC was assessed by haematological analyzer Sysmex K-4500^®^ (Sysmex Polska), and glucose, total cholesterol, HDL, triglycerides, HbA_1_C, CRP, creatinine—by biochemical analyzer Cobas Integra 800^®^ (Roche). Concentration of serum LDL cholesterol was counted according to Friedwald schedule. The following methods were used: enzymatic colorimetric method (total cholesterol, triglycerides, HDL), Jaffe’s method (creatinine), immune turbidmetric method (CRP, HbA_1_C), reference enzymatic method (glucose).

**Fig. 1:**
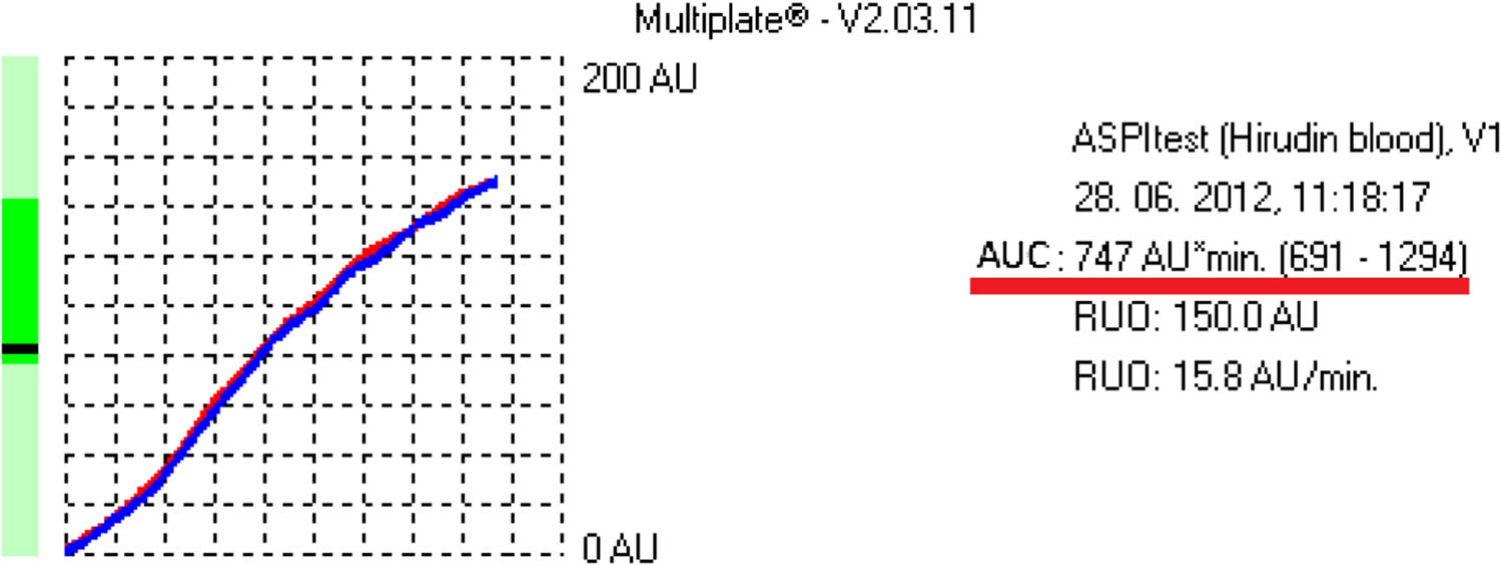
An example of platelet function analysis (ASPI test) with usage of multiple platelet function analyzer. Resistance to ASA was recognized in examined patient

**Fig. 2:**
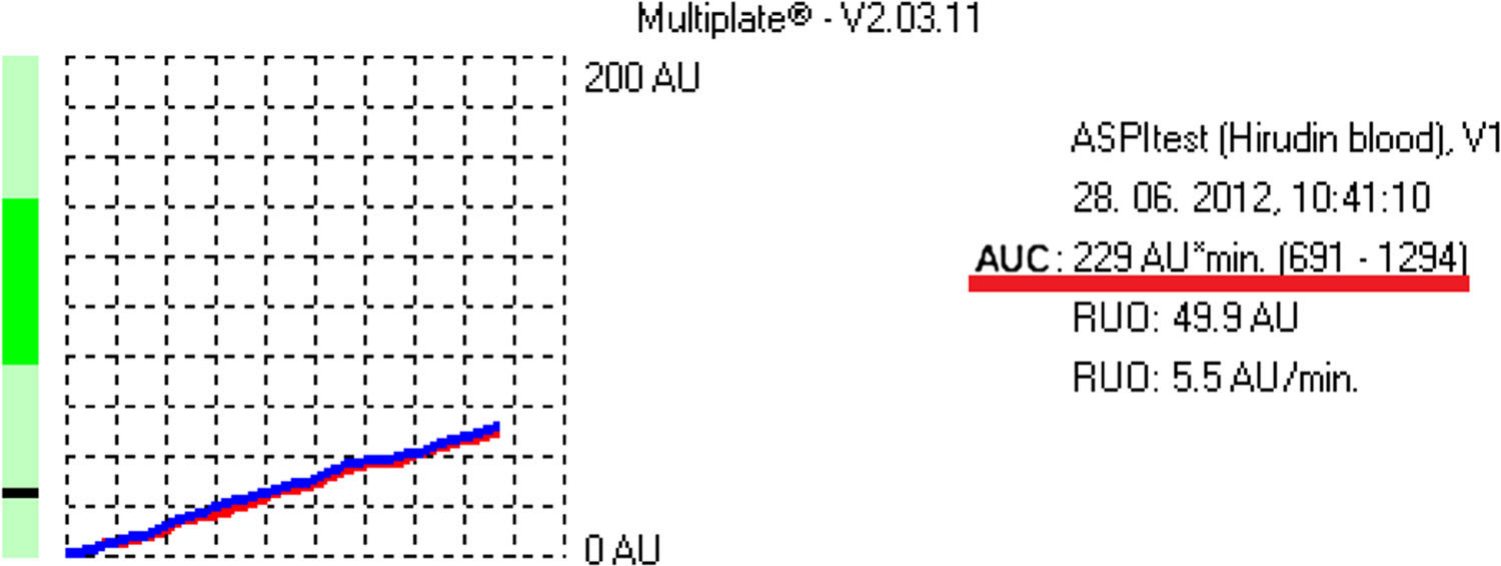
An example of platelet function analysis (ASPI test) with usage of multiple platelet function analyzer. Proper response to ASA was recognized in examined patient

Platelet function test was performed by the method of whole blood impedance aggregometry using multiple platelet function analyzer Multiplate^®^ (Dynabyte Medical, Munich, Germany) [22, 23]. Aggregation was triggered by use of arachidonic acid (ASPItest). The results were expressed as the area under curve (AUC) (Figs. 1, 2).

Based on the manufacturer’s data, it was accepted that in patients who take ASA the value of AUC < 300 indicates aspirin sensitivity (ASA responders), and the value of AUC ≥ 300 indicates aspirin resistance (ASA non-responders) [22–24].

The obtained results underwent statistical analysis using STATISTICA 9.0, Stat Soft Poland. The result was considered statistically significant if obtained significance level was *p* ≤ 0.05. In data description the standard statistical parameters were provided, i.e. the number *N*, arithmetic mean *X*, the standard deviation (SD) and percentages (%). Normal distribution of data was assessed by Shapiro–Wilk normality test. Statistical significance of between-group differences was verified by *U* Mann–Whitney test. Chi-squared test was utilized for comparison of qualified variables. Multiple logistic regression analysis was performed for searching factors associated with AR. Odds ratio (OR) and confidential interval (CI) were calculated for the following factors: age > 60 years, male sex, dose of ASA ≤ 100 mg per day, duration of ASA therapy > 1 year, known duration of diabetes > 5 years, BMI > 25 kg/m^2^, systolic blood pressure ≥ 140 mmHg, diastolic blood pressure ≥ 90 mmHg, heart rate ≥ 80 bpm, presence of each cardiovascular risk factor, each medication, RBC < 4 × 10^6^/μl, RBC > 4 × 10^6^/μl, HGB < 11 g/dl, HGB > 14 g/dl, HCT < 35 %, HCT > 40 %, WBC > 10 × 10^3^/μl, PLT > 300 × 10^3^/μl, HbA_1_C > 6 %, total cholesterol > 5.2 mmol/l, LDL > 3.5 mmol/l, HDL < 1.55 mmol/l, TG > 1.7 mmol/l, CRP > 5 mg/dl, glukoza > 110 mg/dl, kreatynina > 1.3 mg/l.

## Results

The examined group consisted of 96 subjects (48 female and 48 male) with diagnosed diabetes mellitus type 2 (mean age 65.3 ± 8.1, mean known duration of DM 9.9 ± 8.1). 53 patients (55.2 %) were treated with oral hypoglycaemic drugs, 41 (42.7 %)—with insulin, and 2 (2.1 %) were only on the hypoglycaemic diet.

Among them, 51 patients (53.1 %) were sensitive to ASA action (ASA responders) and 45 patients (46.9 %) were resistant to ASA action (ASA non-responders).

Clinical characteristics of the examined patients and the ASA response groups are shown in Table 1. The occurrence of other cardiovascular risk factors, the intake of medications and the results of laboratory tests are shown in Tables 2, 3, and 4.

**Table 1:** Clinical characteristics of all the examined patients and ASA response groups

	All the patients (*N* = 96)	ASA responders (*N* = 51)	ASA non-responders (*N* = 45)	*p* value
Female/male^a^	48 (50 %)/48 (50 %)	25 (49 %)/26 (51 %)	23 (51 %)/22 (49 %)	NS^#^
Age (years)^b^	65.3 ± 8.1	66.7 ± 7	63.9 ± 7.9	NS^##^
ASA (75 mg)/ASA (150 mg)^a^	55 (57 %)/41 (43 %)	29 (57 %)/22 (43 %)	26 (58 %)/19 (42 %)	NS^#^
Duration of ASA therapy (years)^b^	4.9 + 3.6	5.7 ± 3.9	3.9 ± 2.9	0.010^##^
Known duration of diabetes (years)^b^	9.9 ± 8.1	10.8 ± 8.1	8.6 ± 7.9	NS^##^
BMI (kg/m^2^)^b^	30.1 ± 4.6	30.2 ± 4.9	30.1 ± 4.4	NS^##^
HR (bpm)^b^	74.9 ± 7.8	74.1 ± 7.5	75.7 ± 8.0	NS^##^
SBP (mmHg)^b^	134.4 ± 13.7	134.6 ± 12.5	134.1 ± 14.9	NS^##^
DBP (mmHg)^b^	78.8 ± 7.9	77.6 ± 8.3	80.0 ± 7.5	NS^##^

*BMI* body mass index, *ASA* acetylsalicylic acid, *HR* heart rate, *SBP* systolic blood pressure, *DBP* diastolic blood pressure

^#^ *χ* ^2^ test

^##^ *U* Mann–Whitney test

^a^Data presented as *N* (%)

^b^Data presented as mean ± SD

**Table 2:** Cardiovascular risk factors present in all the examined patients and ASA response groups

Risk factor	All the patients (*N* = 96)	ASA responders (*N* = 51)	ASA non-responders (*N* = 45)	*p* value*
Arterial hypertension	90 (93.8 %)	48 (94.1 %)	42 (93.3 %)	NS
Coronary heart disease	50 (52.1 %)	28 (54.9 %)	22 (48.9 %)	NS
Previous myocardial infarct	19 (19.8 %)	10 (19.6 %)	9 (20 %)	NS
Previous stroke	30 (31.3 %)	16 (53.3 %)	14 (46.7 %)	NS
Dyslipidaemia	85 (88.5 %)	44 (86.3 %)	41 (91.1 %)	NS
Atrial fibrilation	23 (23.9 %)	12 (23.5 %)	11 (24.4 %)	NS
Current smoking	15 (15.6 %)	4 (7.8 %)	11 (24.4 %)	0.030
Overweight or obesity**	83 (86.5 %)	44 (86.3 %)	39 (86.7 %)	NS

In brackets frequencies of occurrence of risk factors in each group are shown, *N* (%)

* *χ* ^2^ test

** BMI > 25 kg/m^2^

**Table 3:** Laboratory results (mean ± SD) in all the examined patients and ASA response groups

Laboratory results	All the patients (*N* = 96)	ASA responders (*N* = 51)	ASA non-responders (*N* = 45)	*p* value*
RBC (10^6^/μl)	4.7 ± 1.3	4.5 ± 0.5	4.9 ± 1.9	NS
HCT (%)	40.2 ± 5.4	38.8 ± 6.2	41.6 ± 3.9	0.010
HGB (g/dl)	13.6 ± 1.5	13.3 ± 1.4	13.9 ± 1.6	NS
WBC (10^3^/μl)	7.1 ± 2.2	6.6 ± 1.9	7.7 ± 2.5	0.030
PLT (10^3^/μl)	240.5 ± 66.6	225.1 ± 61.6	257.3 ± 68.4	0.050
Total cholesterol (mmol/l)	4.8 ± 1.3	4.6 ± 1.2	5.2 ± 1.3	0.020
LDL (mmol/l)	3.1 ± 1.2	2.9 ± 1.1	3.5 ± 1.2	0.005
HDL (mmol/l)	1.3 ± 0.4	1.4 ± 0.4	1.3 ± 0.4	NS
TG (mmol/l)	1.6 ± 1.4	1.5 ± 0.7	1.7 ± 0.9	NS
Fasting glucose (mg/dl)	140 ± 60	142.1 ± 70.5	139.1 ± 46.7	NS
HbA_1_C (%)	7.7 ± 6.2	7.9 ± 8.4	7.3 ± 1.7	NS
CRP (mg/l)	3.2 ± 5.7	3.3 ± 5.2	3.1 ± 6.3	NS
Creatinine (mg/l)	0.87 ± 0.2	0.8 ± 0.2	0.9 ± 0.3	NS

*RBC* red blood cells, *HCT* haematocrit, *HGB* haemoglobin, *WBC* white blood cells, *PLT* platelets, *LDL* low density lipoproteins, *HDL* high density lipoproteins, *TG* triglycerides, *CRP* C-reactive protein, *HbA*_*1*_ *C* glycated haemoglobin A_1_C

* *U* Mann–Whitney test

**Table 4:** Additional medications used in all the examined patients and ASA response groups

Medication	All the patients (*N* = 96)	ASA responders (*N* = 51)	ASA non-responders (*N* = 45)	*p* value*
Oral hypoglycaemics	53 (55.2 %)	28 (54.9 %)	25 (55.6 %)	NS
Insulin	41 (42.7 %)	22 (43.1 %)	19 (42.2 %)	NS
Diuretics	29 (30.2 %)	19 (37.3 %)	10 (22.2 %)	NS
ACE inhibitors	73 (76.0 %)	36 (70.6 %)	37 (87.2 %)	NS
ARBs	13 (13.5 %)	8 (15.7 %)	5 (11.1 %)	NS
Calcium antagonists	28 (29.2 %)	18 (35.3 %)	10 (22.2 %)	NS
Beta-blockers	50 (52.1 %)	24 (47.1 %)	26 (57.8 %)	NS
Nitrates	18 (18.8 %)	7 (13.7 %)	11 (24.4 %)	NS
Statins	67 (69.8 %)	36 (70.6 %)	31 (68.9 %)	NS
Fibrates	7 (7.3 %)	3 (5.9 %)	4 (8.9 %)	NS
PPIs	13 (13.5 %)	6 (11.8 %)	7 (15.6 %)	NS

In brackets, percentages of patients in each group taking the appropriate medication are presented

*ACE* inhibitors angiotensin converting enzyme inhibitor, *ARBs* angiotensin II receptor blockers, *PPIs* proton-pump inhibitors

* *χ* ^2^ test

No association was found between platelet aggregation and the gender, age, the dose of ASA, known duration of diabetes, BMI, heart rate, mean systolic and diastolic blood pressure. Patients resistant to ASA were treated shorter with ASA than ASA responders (*p* = 0.010). No association was found between the occurrence of ASA resistance and the risk factors found in patients except for current smoking (Table 2).

The mean total cholesterol (*p* = 0.020), LDL concentration (*p* = 0.005), HCT (*p* = 0.010), WBC (*p* = 0.030), and PLT (*p* = 0.050) were significantly higher in ASA non-responders compared to ASA responders. No association was found between the aggregation parameters and the results of the other laboratory tests (Table 3).

No statistically significant association was found between the intake of different medications and the occurrence of ASA resistance (Table 4).

Multiple logistic regression analysis revealed the factors associated with AR: history of current smoking (OR 3.79, CI 1.08–13.3, *p* = 0.040), and LDL concentration higher than 3.5 mmol/l (OR 5.58, CI 1.26–24.8, *p* = 0.020).

## Discussion

The results of some experimental studies show the significant influence of hyperglycaemia on platelet function since it results in increased prothrombotic condition and proinflammatory functions promoting atherosclerosis and acute vascular episodes by the induction of platelet activation and expression of tissue factor in monocytes. Hyperglycaemia can also be related to the decreased sensitivity to ASA [9, 25, 26]. In our study we did not observe the influence of poor glycaemic control on incidence of AR in diabetic patients. We neither noticed association between fasting blood glucose nor HBA_1_C level and platelet function. In the literature the results concerning the phenomenon of AR and glycaemic control in the diabetic patients are conflicting. Some authors, similar to our results, did not observe association between AR and parameters of glycaemic control [20, 21]. On the other hand, Ertugrul et al. investigated 108 diabetic patients and found that AR correlated positively with fasting blood glucose and HbA_1_C. In this study the patients with poor glycaemic control (HbA1C > 7 %) had significantly higher AR [16]. Association of AR with HbA_1_C was also observed by Cohen et al. [19] and Watala et al. [17].

We noticed association between AR and lipid disorders in diabetic patients. ASA non-responders had significantly higher concentration of serum total cholesterol and LDL cholesterol in comparison to ASA responders. It is consistent with our previous results and literature data [13, 26, 27]. Karepov et al. [13] observed the association between the lack of antiaggregation and triglicerydaemia despite ASA intake. Friend et al. [27] noticed ineffective platelet response to ASA in 69 % of examined patients with hyperlipidaemia.

The results of our study did not demonstrate the association between ASA dose (75 or 150 mg) and the AR phenomenon. The other reports concerning this problem are conflicting. Some authors found that patients taking smaller doses of ASA had a greater risk of AR [16, 28, 29]. However, such a relationship was not confirmed by large randomized trials [30].

Similarly to most researchers we found no association between platelet function and the gender of patients [8, 11]. We also did not observe an association between the age and AR what is consistent with many reports [8, 11]. Only a few authors described that the elderly required the administration of higher doses of ASA to achieve antiaggregative effect [9, 31, 32].

Besides, we did not find the association between AR and increased BMI, similarly to Zytkiewicz et al. [11]. Some researchers observed higher percentage of AR in obese patients [13, 33].

We did not observe the association between platelet reactivity and cardiovascular risk factors except for current smoking. In the literature there are some reports about increased AR in people taking ASA and smoking [34–36]. The concentration of 8-izo-PGF2α, the prostaglandin synthesized from arachidonic acid in non-enzymatic process catalyzed by free radicals, is elevated in smokers. This substance increases the platelet response to agonists used in the laboratory tests [37, 38].

The association between AR and the duration of ASA intake was also reported in the literature. The majority of researchers noted a higher percentage of ASA non-responders among patients taking this medication for a long time, but we did not observe such situation [10, 39]. In our study mean time of treatment with ASA was significantly longer in ASA responders than in ASA non-responders. It could be associated with significantly increased concentrations of total and LDL cholesterol, and higher smoking prevalence in ASA non-responders in comparison to ASA responders.

Similarly to other authors, we found association between parameters of CBC and AR [40–42]. ASA non-responders had higher values of HCT, WBC and PLT in comparison with ASA responders.

We did not observe a statistically significant association between AR and other medication taken by examined patients. In the available literature there are reports that resistance to antiplatelet medicine is increased in people treated with NSAIDs (non steroidal anti-inflammatory drugs), CCB (calcium channel blockers), SSRI (selective serotonin reuptake inhibitors), ACE inhibitors, beta-blockers, and PPI (proton pump inhibitors) [9, 43–47]. On the other hand, it is described that the probability of the occurrence of ASA resistance was decreased in people treated with statins, which might be related to the reduction in the cholesterol level [48]. There are no reports regarding interactions between ASA and fibrates, nitrates and angiotensin II receptor blockers.

## Conclusions

Results of our study did not confirm the association between poor glycaemic control in the diabetic patients and the phenomenon of AR. Lipid disorders are associated with AR. Diabetic patients who take ASA and smoke are at greater risk of AR.

## Limitation of the study

As the literature data indicate, patient non-compliance is an important factor which limits ASA effect [49]. In the examined group of diabetic patients, the regularity of ASA intake was determined based on the carefully obtained medical history. Besides, the intake of ASA was personally controlled by one of the authors on the day (24 h) before the study.

AR was determined by only one laboratory method, the whole blood impedance aggregometry using a new-generation multiple platelet function analyzer. In comparison to traditional optic aggregometry by Born, this method is much quicker, less laborious and does not require a special preparation of the blood sample, hence decreasing the risk of the laboratory error. The aggregation was measured by only one agonist, arachidonic acid, as it is suggested for AR detection.

## Abbreviations

ACE: Angiotensin converting enzyme
AR: Resistance to acetylsalicylic acid
ASA: Acetylsalicylic acid
AUC: Area under curve
BP: Blood pressure
CBC: Complete blood count
CCB: Calcium channel blockers
CRP: C-reactive protein
DBP: Diastolic blood pressure
HCT: Haematocrit
HGB: Haemoglobin
NSAID: Non-steroidal anti-inflammatory drugs
PLT: Platelets
PPI: Proton pump inhibitors
RBC: Red blood cells
SCORE: Systematic coronary risk evaluation
SBP: Systolic blood pressure
SSRI: Selective serotonin reuptake inhibitors
WBC: White blood cells
